# Supplementary material for: Distinguishing mechanisms underlying EMT tristability
Source: Cancer Converg. 2017 Nov 1;1(1):2. doi: 10.1186/s41236-017-0005-8 (PMC5876698; doi:10.1186/s41236-017-0005-8)
Supplement: Supplementary file 1 — SI on model formulation and experiments. (DOCX 4967 kb) [file 41236_2017_5_MOESM1_ESM.docx]

**Distinguishing Mechanisms Underlying EMT Tristability**

Dongya Jia, Mohit Kumar Jolly, Satyendra C. Tripathi, Petra Den Hollander, Bin Huang, Mingyang Lu, Muge Celiktas, Esmeralda Ramirez-Peña, Eshel Ben-Jacob, José N. Onuchic, Samir M. Hanash, Sendurai A. Mani, Herbert Levine

**Supplemental Information**

1. **The ternary chimera switch (TCS) model**

The framework of the TCS model (1,2) starts with a detailed description of the transcription factor (TF) – promoter binding/unbinding dynamics and adds the miRNA – mRNA binding/unbinding dynamics. The deterministic equations of a circuit with microRNA ($\mu$), mRNA ($m$) and protein ($B$) are:

$$\frac{d\mu}{dt}=g_{\mu}-mY_{\mu}\left( \mu\right)-k_{\mu}\mu(eq. 1.1)$$

$$\frac{dm}{dt}=g_{m}-mY_{m}\left( \mu\right)-k_{m}m (eq. 1.2)$$

$$\frac{dB}{dt}=g_{B}mL\left( \mu\right)-k_{B}B (eq. 1.3)$$

where $g_{\mu}$, $g_{m}$ and $g_{B}$ are the production terms of $\mu$, $m$ and $B$ respectively, which are the function of corresponding regulating transcriptional factors, $k_{\mu}$, $k_{m}$ and $k_{B}$ are the innate degradation rates of $\mu$, $m$ and $B$ respectively. The functions $Y_{\mu}\left( \mu\right)$ and $Y_{m}\left( \mu\right)$ represent the degradation of $\mu$ and $m$ respectively due to the formation of microRNA-mRNA complex. The function $L\left( \mu\right)$ represents the inhibition of the translation of $m$ due to microRNA.

microRNA can bind to the 3’ UTR of an mRNA to form a microRNA-mRNA complex, by which microRNA can inhibit the translation of mRNA and/or actively degrades mRNA and meanwhile microRNA can be degraded or recycled. The TCS model establishes a theoretical framework for microRNA-mRNA interaction to capture all these features.

Suppose the 3’UTR of a mRNA ($m$) has $n$ microRNA ($\mu$) binding sites. There are then a total of $\left( n+1 \right)$ possible configurations of mRNA, each of which binds $i$ (0$\leq i\leq n$) microRNA(s). Since the length of a microRNA is about 22nt and the length of the seed sequence on mRNA identified by microRNA is around 7-8nt, the binding of microRNA to different binding sites on mRNA is considered to be independent. In addition, the binding/unbinding of microRNA to mRNA is considered to be at equilibrium since it is assumed to be much faster compared with the production/degradation of molecules. When $i$ microRNA(s) bind(s) to $i$ binding sites of 3’UTR of mRNA, the concentration of mRNA ($m_{i}$, representing mRNA in the configuration with $i$ microRNA binding) obeys:

$$r_{\mu+}\mu\left[ m_{i} \right]=r_{\mu-}\left[ m_{i+1} \right] (eq. 1.4)$$

where $r_{\mu+}$ represents the binding rate and $r_{\mu-}$ represents the unbinding rate. $\mu$ represents the concentration of microRNA. $(eq. 1.4)$ can be rewritten as:

$$\left[ m_{i} \right]=\left( \frac{\mu}{\mu_{0}} \right)^{i}\left[ m_{0} \right] (eq. 1.5)$$

where $\mu_{0}=\frac{r_{\mu-}}{r_{\mu+}}$.

All terms $\left[ m_{i} \right]$ should satisfy $\sum_{i=0}^{n} C_{n}^{i}\left[ m_{i} \right]=m$, where $m$ represents the total concentration of mRNA, $C_{n}^{i}=\frac{n!}{i!\left( n-i \right)!}$, as there are $C_{n}^{i}$ different mRNA configurations when $i$ out of $n$ binding sites are occupied by microRNAs. So,

$$\left[ m_{i} \right]=mM_{n}^{i}\left( \mu\right) (eq. 1.6)$$

where $M_{n}^{i}\left( \mu\right)=\frac{\left( \frac{\mu}{\mu_{0}} \right)^{i}}{\left( 1+\frac{\mu}{\mu_{0}} \right)^{n}}$.

Thus the total translation rate is

$$mL\left( \mu\right)=\sum_{i=0}^{n} {l_{i}C}_{n}^{i}\left[ m_{i} \right]=m{\sum_{i=0}^{n} {l_{i}C}_{n}^{i}M}_{n}^{i}\left( \mu\right) (eq. 1.7)$$

The total mRNA active degradation rate is

$$mY_{m}\left( \mu\right)=\sum_{i=0}^{n} {\gamma_{mi}C}_{n}^{i}\left[ m_{i} \right]=m{\sum_{i=0}^{n} {\gamma_{mi}C}_{n}^{i}M}_{n}^{i}\left( \mu\right) (eq. 1.8)$$

The total microRNA active degradation rate is

$$mY_{\mu}\left( \mu\right)=\sum_{i=0}^{n} {\gamma_{\mu i}C}_{n}^{i}\left[ m_{i} \right]=m{\sum_{i=0}^{n} {i\gamma_{\mu i}C}_{n}^{i}M}_{n}^{i}\left( \mu\right) (eq. 1.9)$$

where $l_{i}$, $\gamma_{mi}$ and $\gamma_{\mu i}$ represent the individual translation rate, degradation rates of $m$ and $\mu$.

The shifted hill function, which is defined as $H^{S}=\frac{1+\lambda\left( \frac{B}{B_{0}} \right)^{n_{B}}}{1+\left( \frac{B}{B_{0}} \right)^{n_{B}}}$, has been used to represent the transcriptional regulation in the TCS model. In the TCS model, $\lambda$ is the fold change from basal synthesis rate due to the regulation of protein B, $B$ represents the levels of protein B, $B_{0}$ is the threshold and $n_{B}$ represents the number of binding sites. $H^{S+}$ represents the transcriptional activation with the fold change parameter $\lambda>1$ and $H^{S-}$ represents the transcriptional inhibition with the fold change parameter $\lambda<1$. The other details of the formulation of the TCS model can be found in (2).

1. **The cascading bistable switches (CBS) model.**

The CBS model (3) follows the procedure, which is developed in the TCS model, to simulate the miRNA-mRNA interaction. Similarly, the CBS model considers the two mechanisms of regulation of mRNA by miRNA – translational repression and target degradation. In the CBS model, the deterministic equations of a circuit with miRNA ($\mu_{t}$ representing the total level of miRNA and $\mu$ representing the free miRNA without binding to mRNA), mRNA ($m_{t}$ representing the total level of mRNA and $m$ representing the free mRNA without binding to miRNA), miRNA-mRNA complex ($R$) and protein ($B$) are:

$$\frac{d\mu_{t}}{dt}=g_{\mu}-k_{\mu}\mu- k_{R}\left( 1-\lambda\right)R (eq. 2.1)$$

$$\frac{dm_{t}}{dt}=g_{m}-k_{m}m- k_{R}R (eq. 2.2)$$

$$\frac{dR}{dt}=k_{on}\left( \mu_{t}-\mu\right)\left( m_{t}-m \right)-k_{off}R- k_{R}R (eq. 2.3)$$

$$\frac{dB}{dt}=k_{s0}m+k_{s1}R-k_{B}B (eq. 2.4)$$

where $g_{\mu}$ and $g_{m}$ are the innate production rates of miRNA and mRNA respectively, $k_{\mu}$, $k_{B}$, $k_{R}$, and $k_{B}$ are the innate degradation rates of miRNA, mRNA, miRNA-mRNA complex and protein respectively. $k_{on}$ and $k_{off}$ represent the binding and unbinding rates between miRNA and mRNA respectively. $k_{s0}$ and $k_{s1}$ represents the translation rates corresponding to free mRNA and mRNA-miRNA complex respectively. $\lambda$ represents the recycle ratio of miRNA. Positive and negative hill function are used in the CBS model to mimic the transcriptional activation and transcriptional inhibition. The other details about the formulation of the CBS model can be found in (3).

1. **The model formulation of CD44s/ZEB1 feedback loops.**

Here, we replace the direct self-activation of ZEB considered by Lu et al. (2) with the ZEB1/ESRP1/CD44s feedback loop. There are seven components in the miR-200/ZEB1/ESRP1/CD44s circuit - microRNA miR-200 ($\mu_{200}$), ZEB1 mRNA ($m_{Z}$), ZEB1 protein ($Z$), ESRP1 mRNA ($m_{E}$), ESRP1 protein ($E$), CD44v ($C_{V}$), and CD44s ($C_{S}$). The first 5 components have an innate production and degradation rate respectively. Based on experiments that ESRP1 affects the splicing of CD44 and not its absolute levels (4), total levels of CD44 ($C_{T}=C_{S}+C_{V}$) have been considered to be constant, instead what vary are the levels of CD44v and CD44s.

The equation describing the miR-200/ZEB1/ESRP1/CD44s circuit are as follows,

$$\frac{d\mu_{200}}{dt}=g_{\mu_{200}}H^{S-}\left( Z,Z_{\mu_{200}}^{0}{,n}_{Z,\mu_{200}}{,\lambda}_{Z,\mu_{200}} \right)H^{S-}\left( S,S_{\mu_{200}}^{0}{,n}_{S,\mu_{200}}{,\lambda}_{S,\mu_{200}} \right)-m_{Z}Y_{\mu}\left( \mu_{200} \right)-k_{\mu_{200}}\mu_{200} \left( eq. 3.1 \right)$$

$$\frac{{dm}_{Z}}{dt}=g_{m_{Z}}H^{S+}\left( S, S_{0,m_{Z}},n_{S,m_{Z},}\lambda_{S,m_{Z}} \right)H^{S+}\left( C_{s}, C_{s0,m_{Z}},n_{C_{s},m_{Z},}\lambda_{C_{s},m_{Z}} \right)-m_{Z}Y_{m}\left( \mu_{200} \right)-k_{m_{Z}}m_{Z} (eq. 3.2)$$

$$\frac{dZ}{dt}=g_{Z}m_{Z}L\left( \mu_{200} \right)-k_{Z}Z \left( eq. 3.3 \right)$$

$$\frac{{dm}_{E}}{dt}=g_{m_{E}}H^{S-}\left( Z, Z_{0,m_{E}},n_{Z,m_{E},}\lambda_{Z,m_{E}} \right)-k_{m_{E}}m_{E} (eq. 3.4)$$

$$\frac{dE}{dt}=g_{E}m_{E}-k_{E}E (eq. 3.5)$$

$$\frac{dC_{s}}{dt}=C_{T}\frac{1}{1+\left( \frac{E_{C_{s}}}{E_{C_{s},0}} \right)^{n_{E,C_{s}}}} (e.q. 3.6)$$

$$C_{T}=C_{S}+C_{V} (e.q. 3.7)$$

In $(eq. 3.1)$, $g_{\mu_{200}}$ and $k_{\mu_{200}}$ represent the innate production and degradation rates of miR-200 respectively. The shifted Hill functions $H^{S-}\left( Z,Z_{\mu_{200}}^{0}{,n}_{Z,\mu_{200}}{,\lambda}_{Z,\mu_{200}} \right)$ and $H^{S-}\left( S,S_{\mu_{200}}^{0}{,n}_{S,\mu_{200}}{,\lambda}_{S,\mu_{200}} \right)$ represent the transcriptional inhibition of miR-200 by ZEB1 and SNAIL respectively. $Y_{\mu}\left( \mu_{200} \right)$ represents the degradation of miR-200 due to the interaction with ZEB1 mRNA.

In $(eq. 3.2)$, $g_{m_{Z}}$ and $k_{m_{Z}}$ represent the innate production and degradation rates of ZEB1 mRNA respectively. The shifted Hill functions $H^{S+}\left( S, S_{0,m_{Z}},n_{S,m_{Z},}\lambda_{S,m_{Z}} \right)$ $H^{S+}\left( C_{s}, C_{s0,m_{Z}},n_{C_{s},m_{Z},}\lambda_{C_{s},m_{Z}} \right)$ represent the activation of ZEB1 by CD44s and SNAIL respectively. $Y_{m}\left( \mu_{200} \right)$ represents the degradation of ZEB1 mRNA due to the binding of miR-200.

In $(eq. 3.3)$, $g_{Z}$ and $k_{Z}$ represent the innate production and degradation rates of ZEB1 protein respectively. $L\left( \mu_{200} \right)$ represents the translational inhibition of ZEB1 mRNA by miR-200.

In $(eq. 3.4)$, $g_{m_{E}}$ and $k_{m_{E}}$ represent the innate production and degradation rates of ESRP1 mRNA respectively. The shifted Hill function $H^{S-}\left( Z, Z_{0,m_{E}},n_{Z,m_{E},}\lambda_{Z,m_{E}} \right)$ represents the inhibition of ESRP1 by ZEB1.

In $(eq. 3.5)$, $g_{E}$ and $k_{E}$ represent the innate production and degradation rates of ESRP1 protein respectively.

In $(eq. 3.6)$, $C_{T}$ represents the total amount of CD44s and the negative Hill function $\frac{1}{1+\left( \frac{E_{C_{s}}}{E_{C_{s},0}} \right)^{n_{E,C_{s}}}}$ represents the splicing of CD44 by ESRP1.

The relevant parameters can be found in Table S1.

1. **Parameter sensitivity analysis of modeling CD44s/ZEB1 feedback loops**

To understand the robustness of the tristability endowed by the CD44s/ZEB1 feedback loop, we conducted a sensitivity analysis via parameter perturbation, changing one parameter at a time increasing/decreasing its original value by $20\%$. The numbers of binding sites have been considered to be fixed. All other parameters have been varied.

We increased or decreased each parameter by 20% (each case is denoted by an alphanumeric code) and plotted the range of SNAIL levels for which the hybrid E/M phenotype exists (**Figure S1**). The absolute levels of SNAIL that enable the existence of hybrid E/M state increase or decrease for most cases. However, here we focus on the change of the range of SNAIL levels for the existence of the hybrid E/M state, when different parameters are varied.

Compared with the control case (no parameter changed), the range of SNAIL for the existence of E/M state decreases when the production rate of ESRP1 mRNA ($g_{m_{E}}$) is increased (case D1), translation rate for ESRP1 mRNA into ESRP1 protein ($g_{E}$) is increased (case E1), degradation rate for ESRP1 mRNA ($k_{m_{E}}$) or protein ($k_{E}$) is decreased (cases I2, J2), the threshold of ZEB1 levels ($Z_{\mu_{200}}^{0}$) in the shifted Hill function representing inhibition of ZEB1 on miR-200 are decreased (case K2), threshold of ZEB1 levels for shifted Hill function representing inhibition of ESRP1 by ZEB1 ($Z_{m_{E}}^{0}$) is increased (case O1), and the threshold of ESRP1 levels ($E_{C_{s},0}$) for Hill function representing alternative splicing of CD44 (case P2) is decreased (Figure S1B). All these cases, except for case O1, represent cases where the effective ZEB1 levels that can inhibit miR-200 and drive EMT are increased, i.e. the propensity of the cell to undergo EMT is increased. Conversely, the parameter changes that are likely to decrease the effective ZEB1 levels that can induce EMT should enlarge the range of SNAIL levels for which the hybrid E/M state exists. This is indeed observed for cases when the effective ESRP1 levels increase (cases D2, E2, I1, J1) by affecting the innate production ($g_{m_{E}},g_{E}$) or degradation ($k_{m_{E}}, k_{E}$) rates for ESRP1, the threshold of ZEB1 levels ($Z_{\mu_{200}}^{0}$) for shifted Hill function representing inhibition of ZEB1 on miR-200 is increased (case K1), threshold of ZEB1 levels ($Z_{m_{E}}^{0}$) for shifted Hill function representing the inhibition of ESRP1 by ZEB1 is decreased (case O2). Therefore, consistent with our earlier results (2), we find that change in several parameters of miR-200/ZEB1/CD44/ESRP1 circuit that affect ‘effective’ ZEB1 protein levels, can affect the range of SNAIL levels for which the hybrid E/M state exists. Thus, **Figure S1** shows that for most parameter changes (either increase or decrease), the region of SNAIL levels for which the hybrid E/M exists doesn’t change much, which demonstrate the tristability enabled by the CD44s/ZEB1 feedback loop is quite robust.

1. **The formulation of the TCS-FOXC2 model.**

FOXC2 can be up-regulated by SNAIL and directly activate the transcription of ZEB1 (5,6). Therefor FOXC2 is included to the TCS model as the target of SNAIL and upstream regulator of ZEB1. The deterministic equations for microRNA miR-200 ($\mu_{200}$), ZEB1 mRNA ($m_{Z}$), ZEB1 protein ($Z$), miR-34 ($\mu_{34}$), SNAIL mRNA ($m_{S}$), SNAIL protein ($S$), FOXC2 mRNA $\left( m_{F} \right)$and FOXC2 protein $\left( F \right)$in the miR-34/SNAIL/miR-200/ZEB1/FOXC2 circuit are:

$$\frac{d\mu_{200}}{dt}=g_{\mu_{200}}H^{S-}\left( Z,Z_{\mu_{200}}^{0}{,n}_{Z,\mu_{200}}{,\lambda}_{Z,\mu_{200}} \right)H^{S-}\left( S,S_{\mu_{200}}^{0}{,n}_{S,\mu_{200}}{,\lambda}_{S,\mu_{200}} \right)-m_{Z}Y_{\mu}\left( \mu_{200} \right)-k_{\mu_{200}}\mu_{200} \left( eq. 5.1 \right)$$

$$\frac{{dm}_{Z}}{dt}=g_{m_{Z}}H^{S+}\left( Z,Z_{m_{Z}}^{0}{,n}_{Z,m_{Z}}{,\lambda}_{Z,m_{Z}} \right)H^{S+}\left( F,F_{m_{Z}}^{0}{,n}_{F,m_{Z}}{,\lambda}_{F,m_{Z}} \right)-m_{Z}Y_{m}\left( \mu_{200} \right)-k_{m_{Z}}m_{Z} \left( eq. 5.2 \right)$$

$$\frac{dZ}{dt}=g_{Z}m_{Z}L\left( \mu_{200} \right)-k_{Z}Z \left( eq. 5.3 \right)$$

$$\frac{d\mu_{34}}{dt}=g_{\mu_{34}}H^{S-}\left( Z,Z_{\mu_{34}}^{0}{,n}_{Z,\mu_{34}}{,\lambda}_{Z,\mu_{34}} \right)H^{S-}\left( S,S_{\mu_{34}}^{0}{,n}_{S,\mu_{34}}{,\lambda}_{S,\mu_{34}} \right)-m_{S}Y_{\mu}\left( \mu_{34} \right)-k_{\mu_{34}}\mu_{34} \left( eq. 5.4 \right)$$

$$\frac{{dm}_{S}}{dt}=g_{m_{S}}H^{S-}\left( S,S_{m_{S}}^{0}{,n}_{S,m_{S}}{,\lambda}_{S,m_{S}} \right)H^{S+}\left( S_{A},{S_{A}}_{m_{S}}^{0}{,n}_{S_{A},m_{S}}{,\lambda}_{S_{A},m_{S}} \right)-m_{S}Y_{m}\left( \mu_{34} \right)-k_{m_{s}}m_{s} \left( eq. 5.5 \right)$$

$$\frac{dS}{dt}=g_{S}m_{S}L\left( \mu_{34} \right)-k_{S}S \left( eq. 5.6 \right)$$

$$\frac{{dm}_{F}}{dt}=g_{m_{F}}H^{S+}\left( S, S_{0,m_{F}},n_{S,m_{F},}\lambda_{S,m_{F}} \right)H^{S-}\left( S_{I}, S_{I, m_{F},}^{0},n_{S_{I},m_{F},}\lambda_{S_{I},m_{F}} \right)-k_{m_{F}}m_{F} (eq. 5.7)$$

$$\frac{dF}{dt}=g_{F}m_{F}-k_{F}F (eq. 5.8)$$

In $\left( eq. 5.2 \right)$, The shifted Hill function $H^{S+}\left( F,F_{m_{Z}}^{0}{,n}_{F,m_{Z}}{,\lambda}_{F,m_{Z}} \right)$ represents the transcriptional activation of ZEB1 by FOXC2. The explanation of other terms in $\left( eq. 5.1 \right)$, $\left( eq. 5.2 \right)$ and $\left( eq. 5.3 \right)$ have already been talked in SI section 3 - $\left( eq. 3.1 \right)$, $\left( eq. 3.2 \right)$ and $\left( eq. 3.3 \right)$.

In $(eq. 5.4)$, $g_{\mu_{34}}$ and $k_{\mu_{34}}$ represent the innate production and degradation rates of miR-34 respectively. The shifted Hill functions $H^{S-}\left( Z,Z_{\mu_{34}}^{0}{,n}_{Z,\mu_{34}}{,\lambda}_{Z,\mu_{34}} \right)$ and $H^{S-}\left( S,S_{\mu_{34}}^{0}{,n}_{S,\mu_{34}}{,\lambda}_{S,\mu_{34}} \right)$ represent the transcriptional inhibition of miR-34 by ZEB1 and SNAIL respectively. $Y_{\mu}\left( \mu_{34} \right)$ represents the degradation of miR-34 due to the interaction with SNAIL mRNA.

In $(eq. 5.5)$, $g_{m_{S}}$ and $k_{m_{S}}$ represent the innate production and degradation rates of SNAIL mRNA respectively. The shifted Hill functions $H^{S-}\left( S,S_{m_{S}}^{0}{,n}_{S,m_{S}}{,\lambda}_{S,m_{S}} \right)$ and $H^{S+}\left( S_{A},{S_{A}}_{m_{S}}^{0}{,n}_{S_{A},m_{S}}{,\lambda}_{S_{A},m_{S}} \right)$ represent the self-inhibition of SNAIL and activation of SNAIL by external EMT-inducing signal $S_{A}$ respectively. $Y_{m}\left( \mu_{34} \right)$ represents the degradation of SNAIL mRNA due to the binding of miR-34.

In $(eq. 5.6)$, $g_{S}$ and $k_{S}$ represent the innate production and degradation rates of SNAIL protein respectively. $L\left( \mu_{34} \right)$ represents the translational inhibition of SNAIL mRNA by miR-34.

In $(eq. 5.7)$, $g_{m_{F}}$ and $k_{m_{F}}$ represent the innate production and degradation rates of FOXC2 mRNA respectively. The shifted Hill functions $H^{S+}\left( S, S_{0,m_{F}},n_{S,m_{F},}\lambda_{S,m_{F}} \right)$ and $H^{S-}\left( S_{I}, S_{I, m_{F},}^{0},n_{S_{I},m_{F},}\lambda_{S_{I},m_{F}} \right)$ represent the activation of FOXC2 by SNAIL and the inhibition of FOXC2 by external inhibitory signal $S_{I}$ respectively.

In $(eq. 5.8)$, $g_{F}$ and $k_{F}$ represent the innate production and degradation rates of SNAIL protein respectively. The relevant parameters have been listed in Table S2.

1. **The formulation of the CBS-FOXC2 model.**

In this section, we follow the same terminology for different components as used in (3). The deterministic equation for SNAIL mRNA (${[snail1]}_{t}$ representing the total level of SNAIL mRNA and $[snail1]$ representing the free SNAIL mRNA without binding to miR-34), miR-34 (${[miR34]}_{t}$ representing the total level of miR-34 and $[miR34]$ representing the free SNAIL mRNA without binding to miR-34), SNAIL protein ($\left[ SNAIL1 \right]$), ZEB1 mRNA (${[zeb]}_{t}$ representing the total level of ZEB1 mRNA and $[zeb]$ representing the free ZEB1 mRNA without binding to miR-200), ZEB1 protein ($\left[ ZEB \right]$), FOXC2 mRNA ($\left[ foxc2 \right]_{t}$ representing the total levels of FOXC2 mRNA),FOXC2 protein $\left[ FOXC2 \right]$, epithelial marker ($\left[ E\_marker \right]$) and mesenchymal marker ($\left[ M\_marker \right]$) are:

$$\frac{{d\left[ snail1 \right]}_{t}}{dt}={k0}_{snail}+k_{snail}*\frac{\left( \frac{\left[ TGF \right]_{t}}{J_{snail0}} \right)^{2}}{1+\left( \frac{\left[ TGF \right]_{t}}{J_{snail0}} \right)^{2}}*\frac{1}{1+\left( \frac{\left[ SNAIL1 \right]_{t}}{J_{snail1}} \right)^{2}}-{kd}_{snail}*\left[ snail1 \right]-{kd}_{SR}*\left[ SR \right] (eq. 6.1)$$

$$\frac{{d\left[ miR34 \right]}_{t}}{dt}={k0}_{34}+k_{snail}*\frac{k_{34}}{1+\left( \frac{\left[ SNAIL1 \right]}{{J1}_{34}} \right)^{2}+\left( \frac{\left[ ZEB \right]}{{J2}_{34}} \right)^{2}}-{kd}_{34}*\left[ miR34 \right]-{\left( 1-\lambda s \right)kd}_{SR1}*\left[ SR \right] (eq. 6.2)$$

$$\frac{d\left[ SNAIL1 \right]}{dt}=k_{SNAIL}*\left[ snail1 \right]-{kd}_{SNAIL}*\left[ SNAIL1 \right] (eq. 6.3)$$

$$\left[ miR34 \right]=\left[ miR34 \right]_{t}-\left[ SR \right] (eq. 6.4)$$

$$\left[ snail1 \right]=\left[ snail1 \right]_{t}-\left[ SR \right] (eq. 6.5)$$

$$\left[ SR \right]=K_{S}*\left[ snail1 \right]*\left[ miR34 \right] (eq. 6.6)$$

$$\left[ TGF \right]_{t}=\left[ TGF \right]+\left[ TGF0 \right] (eq. 6.7)$$

$$\frac{{d\left[ zeb \right]}_{t}}{dt}={k0}_{zeb}+k_{zeb}*\frac{\left( \frac{\left[ FOXC2 \right]}{J_{zebf}} \right)^{2}}{1+\left( \frac{\left[ FOXC2 \right]}{J_{zebf}} \right)^{2}}-{kd}_{zeb}*\left[ zeb \right]-\sum_{i=1}^{5} {kd}_{{ZR}_{i}}*C_{5}^{i}*\left[ {ZR}_{i} \right] (eq.6.8)$$

$$\frac{d\left[ ZEB \right]}{dt}=k_{ZEB}*\left[ zeb \right]-{kd}_{ZEB}*\left[ ZEB \right] (eq. 6.9)$$

$$\frac{{d\left[ miR200 \right]}_{t}}{dt}={k0}_{200}+k_{200}*\frac{1}{1+\left( \frac{\left[ SNAIL1 \right]}{{J1}_{200}} \right)^{3}+\left( \frac{\left[ ZEB \right]}{{J2}_{200}} \right)^{2}}-{kd}_{200}*\left[ miR200 \right]-{\sum_{i=1}^{5} {\left( 1-\lambda_{i} \right)*kd}_{{ZR}_{i}}*C_{5}^{i}*i*\left[ {ZR}_{i} \right]-\left( 1-\lambda_{TR} \right)kd}_{TR}*\left[ TR \right] (eq. 6.10)$$

$$\left[ {ZR}_{i} \right]=K_{i}*\left[ miR200 \right]*\left[ {ZR}_{i-1} \right] \left( i=1\ldots5 \right) (eq. 6.11)$$

$$\left[ zeb \right]=\left[ zeb \right]_{t}-\sum_{i=1}^{5} C_{5}^{i}*\left[ {ZR}_{i} \right] (eq. 6.12)$$

$$\left[ miR200 \right]=\left[ miR200 \right]_{t}-\sum_{i=1}^{5} C_{5}^{i}*\left[ {ZR}_{i} \right] (eq. 6.13)$$

$$\frac{{d\left[ tgf \right]}_{t}}{dt}=k_{tgf}-{kd}_{tgf}\left[ tgf \right]-{kd}_{TR}*\left[ TR \right] (eq. 6.14)$$

$$\frac{d\left[ TGF \right]}{dt}=k_{TGF}*\left[ tgf \right]-{kd}_{TGF}\left[ TGF \right] (eq. 6.15)$$

$$\left[ tgf \right]=\left[ tgf \right]_{t}-\left[ TR \right] (eq. 6.16)$$

$$\left[ TR \right]=K_{TGF}*\left[ miR200 \right]*\left[ tgf \right] (eq. 6.17)$$

$$\frac{{d\left[ foxc2 \right]}_{t}}{dt}=k_{{0m}_{F}}+k_{m_{F}}*\frac{\left( \frac{\left[ SNAIL1 \right]}{J_{foxc20}} \right)}{1+\left( \frac{\left[ SNAIL1 \right]}{J_{foxc20}} \right)}*\frac{\left( \frac{\left[ S_{I} \right]}{J_{foxc21}} \right)}{1+\left( \frac{\left[ S_{I} \right]}{J_{foxc21}} \right)}-{kd}_{foxc2}*\left[ foxc2 \right]_{t} (eq. 6.18)$$

$$\frac{d\left[ FOXC2 \right]}{dt}=k_{FOXC2}*\left[ foxc2 \right]_{t}-{kd}_{FOXC2}*\left[ FOXC2 \right] (eq. 6.19)$$

$$\frac{d\left[ E\_marker \right]}{dt}=k_{e0}+k_{e1}*\frac{1}{1+\left( \frac{\left[ SNAIL \right]}{J_{e1}} \right)^{2}}*k_{e2}\frac{1}{1+\left( \frac{\left[ ZEB \right]}{J_{e2}} \right)^{2}}-{kd}_{e}*\left[ E_{marker} \right] (eq. 6.20)$$

$$\frac{d\left[ M\_marker \right]}{dt}=k_{m0}+k_{m1}*\frac{\left( \frac{\left[ SNAIL \right]}{J_{e1}} \right)^{2}}{1+\left( \frac{\left[ SNAIL \right]}{J_{e1}} \right)^{2}}*k_{e2}\frac{\left( \frac{\left[ ZEB \right]}{J_{e2}} \right)^{2}}{1+\left( \frac{\left[ ZEB \right]}{J_{e2}} \right)^{2}}-{kd}_{m}*\left[ M_{marker} \right] (eq. 6.21)$$

The detailed description of $\left( eq. 6.1 \right)-(eq. 6.21)$ can be found in () except for $\left( eq. 6.18 \right)$ and $(eq. 6.19)$. In $\left( eq. 6.18 \right)$, $k_{{0m}_{F}}$ represents the innate production rate of FOXC2 mRNA. $k_{m_{F}}$ represents the production rate of FOXC2 mRNA when FOXC2 is regulated by SNAIL and the inhibition signal $S_{I}$. ${kd}_{foxc2}$ represent the innate degradation rate of FOXC2 mRNA. In $\left( eq. 6.19 \right)$, $k_{FOXC2}$ and ${kd}_{FOXC2}$ represent the innate production and degradation rates of FOXC2 protein respectively. Due to the regulation of FOXC2, the equation for ZEB1 mRNA is updated as $\left( eq. 6.8 \right)$, where the transcriptional activation of ZEB1 by FOXC2 is represented by $\frac{\left( \frac{\left[ FOXC2 \right]}{J_{zebf}} \right)^{2}}{1+\left( \frac{\left[ FOXC2 \right]}{J_{zebf}} \right)^{2}}$. The relevant parameters can be found in Table S3.

**Supplementary tables**

|  | **Production rate** | | **Molecules/hour** | | **Degradation rate** | | **/hour** |
| --- | --- | --- | --- | --- | --- | --- | --- |
| miR-200 | $g_{\mu_{200}}$ | | 2100 | | $k_{\mu_{200}}$ | | 0.05 |
| ZEB1 mRNA | $g_{m_{Z}}$ | | 11 | | $k_{m_{Z}}$ | | 0.5 |
| ZEB1 protein | $g_{Z}$ | | 100 | | $k_{Z}$ | | 0.1 |
| ESRP1 mRNA | $g_{m_{E}}$ | | 11 | | $k_{m_{E}}$ | | 0.2 |
| ESRP1 protein | $g_{E}$ | | 300 | | $k_{E}$ | | 0.1 |
| CD44 ($C_{T}$) | 400000 molecules | | | | | | |
|  | **Fold-change** | **Value** | **Number of binding sites** | | **Value** | **Threshold** | **Molecules** |
| Inhibition of miR-200 by ZEB1 | $\lambda_{Z,\mu_{200}}$ | 0.1 | $n_{Z,\mu_{200}}$ | | 3 | $Z_{\mu_{200}}^{0}$ | 220000 |
| Inhibition of miR-200 by SNAIL | $\lambda_{S,\mu_{200}}$ | 0.1 | $n_{S,\mu_{200}}$ | | 2 | $S_{\mu_{200}}^{0}$ | 180000 |
| Activation of ZEB1  by SNAIL | $\lambda_{S,m_{Z}}$ | 10 | $n_{S,m_{Z}}$ | | 2 | $S_{m_{Z}}^{0}$ | 180000 |
| $\mu_{0}$ | 10000 | | | | | | |
| $n_{\mu200}$ | 6 | | | | | | |
| Inhibition of ESRP1 by ZEB1 | $\lambda_{Z,m_{E}}$ | 0.1 | | $n_{Z,m_{E}}$ | 2 | $Z_{0,m_{E}}$ | 20000 |
| Activation of ZEB1 by CD44s | $\lambda_{C_{s},m_{Z}}$ | 10 | | $n_{C_{s},m_{Z}}$ | 1 | $C_{s0,m_{Z}}$ | 20000 |
| Splicing of CD44s by ESRP1 | -- | -- | | $n_{E,C_{s}}$ | 2 | $E_{C_{s},0}$ | 15000 |

**Table S1. Parameters used in the modeling of the CD44s/ZEB1 feedback loop.**

|  | **Production rate** | | | **(molecules/hour)** | | **Degradation rate** | | | **(/hour)** | |
| --- | --- | --- | --- | --- | --- | --- | --- | --- | --- | --- |
| miR-200 | $g_{\mu_{200}}$ | | | 2100 | | $k_{\mu_{200}}$ | | | 0.05 | |
| ZEB1 mRNA | $g_{m_{Z}}$ | | | 11 | | $k_{m_{Z}}$ | | | 0.5 | |
| ZEB1 protein | $g_{Z}$ | | | 100 | | $k_{Z}$ | | | 0.1 | |
| miR-34 | $g_{\mu_{34}}$ | | | 1350 | | $k_{\mu_{34}}$ | | | 0.05 | |
| SNAIL mRNA | $g_{m_{S}}$ | | | 90 | | $k_{m_{S}}$ | | | 0.5 | |
| SNAIL protein | $g_{S}$ | | | 100 | | $k_{S}$ | | | 0.125 | |
| FOXC2 mRNA | $g_{m_{F}}$ | | | 110 | | $k_{m_{F}}$ | | | 0.5 | |
| FOXC2 protein | $g_{F}$ | | | 160 | | $k_{F}$ | | | 0.1 | |
|  | **Fold-change** | **Value** | | **Number of binding sites** | | **Value** | **Threshold** | | **(molecules)** | |
| Inhibition of miR-200 by ZEB1 | $\lambda_{Z,\mu_{200}}$ | 0.1 | | $n_{Z,\mu_{200}}$ | | 3 | $Z_{\mu_{200}}^{0}$ | | 220000 | |
| ZEB1 self-activation | $\lambda_{Z,m_{Z}}$ | 7.5 | | $n_{Z,m_{Z}}$ | | 2 | $Z_{m_{Z}}^{0}$ | | 25000 | |
| Inhibition of miR-200 by SNAIL | $\lambda_{S,\mu_{200}}$ | 0.1 | | $n_{S,\mu_{200}}$ | | 2 | $S_{\mu_{200}}^{0}$ | | 180000 | |
| Activation of ZEB1  by SNAIL | $\lambda_{S,m_{Z}}$ | 10 | | $n_{S,m_{Z}}$ | | 2 | $S_{m_{Z}}^{0}$ | | 180000 | |
| Inhibition of miR-34 by SNAIL | $\lambda_{S,\mu_{34}}$ | 0.1 | | $n_{S,\mu_{34}}$ | | 1 | $S_{\mu_{34}}^{0}$ | | 300000 | |
| SNAIL self-inhibition | $\lambda_{S,m_{S}}$ | 0.1 | | $n_{S,m_{S}}$ | | 1 | $S_{m_{S}}^{0}$ | | 200000 | |
| Activation of FOXC2 by SNAIL | $\lambda_{S,m_{F}}$ | 7.5 | | $n_{S,m_{F}}$ | | 1 | $S_{m_{F}}^{0}$ | | 200000 | |
| Activation of ZEB1 by FOXC2 | $\lambda_{F,m_{Z}}$ | 7.5 | | $n_{F,m_{Z}}$ | | 1 | $F_{m_{Z}}^{0}$ | | 200000 | |
| Activation of SNAIL by EMT-inducing signal $S_{A}$ | $\lambda_{S_{A},m_{S}}$ | 10 | | $n_{S_{A},m_{Z}}$ | | 1 | ${S_{A}}_{m_{S}}^{0}$ | | 50000 | |
| Inhibition of FOXC2 by inhibition signal $S_{I}$ | $\lambda_{S_{I},m_{F}}$ | 0.1 | | $n_{S_{I},m_{F}}$ | | 2 | ${S_{I}}_{m_{F}}^{0}$ | | 100000 | |
| $\mu_{0}$ | 10000 | | $n_{\mu200}$ | | 6 | | | $g_{V}$ | | 1000 |

**Table S2. Parameters used in the TCS-FOXC2 model.**

| **Parameter** | **Value** | **Parameter** | **Value** |
| --- | --- | --- | --- |
| **SNAIL1/miR-34 module** | | | |
| ${k0}_{snail}$ | 0.0006 uM/hr | ${k0}_{34}$ | 0.0012 uM/hr |
| $k_{snail}$ | 0.05 uM/hr | $k_{34}$ | 0.012 uM/hr |
| $J_{snail0}$ | 0.62 nM | ${J1}_{34}$ | 0.15 uM |
| $J_{snail1}$ | 0.67 uM | ${J2}_{34}$ | 0.36 uM |
| ${kd}_{snail}$ | 0.09/hr | ${kd}_{34}$ | 0.035/hr |
| ${kd}_{SR}$ | 0.9/hr | $K_{S}$ | 100 /uM |
| $k_{SNAIL}$ | 17 uM/hr | $\lambda s$ | 0.5 |
| $k_{SNAIL}$ | 1.66 /hr |  |  |
| **ZEB1/miR-200 module** | | | |
| ${k0}_{zeb}$ | 0.003 uM/hr | ${k0}_{200}$ | 0.0002 uM/hr |
| $k_{zeb}$ | 0.06 uM/hr | $k_{200}$ | 0.02 uM/hr |
| $J_{zeb}$ | 3.5 nM | ${J1}_{200}$ | 3.25 uM |
| ${kd}_{zeb}$ | 0.09 /hr | ${J2}_{200}$ | 0.2 uM |
| ${kd}_{{ZR}_{i}}$ | 0.09/hr | ${kd}_{200}$ | 0.035/hr |
| $k_{ZEB}$ | 17 uM/hr | $K_{i}$ | 10 /uM |
| ${kd}_{ZEB}$ | 1.66 /hr | $\lambda_{i}$ | 0.5 |
| **FOXC2 module** | | | |
| $k_{0m_{F}}$ | 0.0006 uM/hr | ${kd}_{foxc2}$ | 0.09 uM/hr |
| $k_{m_{F}}$ | 0.042 uM/hr | ${kd}_{FOXC2}$ | 1.66 uM/hr |
| $k_{FOXC2}$ | 17 uM/hr | $J_{zebf}$ | 3.5 uM |
| $J_{foxc20}$ | 3.5 uM | $J_{foxc21}$ | 0.62 uM |
| **TGF-beta module** | | | |
| $k_{tgf}$ | 0.05 uM/hr | $k_{TGF}$ | 1.6 uM/hr |
| ${kd}_{tgf}$ | 0.1 uM/hr | ${kd}_{TGF}$ | 1 /hr |
| ${kd}_{TR}$ | 0.9 /hr | $K_{TGF}$ | 20 uM |
| $\lambda_{TR}$ | 0.8 |  |  |
| **Markers module** | | | |
| $k_{e0}$ | 0.01 uM/hr | $k_{m0}$ | 0.01 uM/hr |
| $k_{e1}$ | 0.15 uM/hr | $k_{m1}$ | 0.1 uM/hr |
| $k_{e2}$ | 0.05 uM/hr | $k_{m2}$ | 0.06 uM/hr |
| $J_{e1}$ | 0.2 uM | $J_{m1}$ | 0.2 uM |
| $J_{e2}$ | 0.5 uM | $J_{m2}$ | 0.5 uM |
| ${kd}_{e}$ | 0.05 /hr | ${kd}_{m}$ | 0.05 /hr |

**Table S3. Parameters used in the CBS-FOXC2 model.**

**Supplementary figures**

**Figure S1. Bifurcation diagram of the miR-200/ZEB1 circuit in response to SNAIL levels without ZEB1 self-activation.** (A) The miR-200/ZEB1 circuit without ZEB1 self-activation. (B) Bifurcation diagram of the ZEB1 mRNA levels in response to SNAIL levels when $g_{m_{Z}}=11$ molecules/hour (Left panel) and $g_{m_{Z}}=80$ molecules/hour. When the basal production rate of ZEB1 mRNA ($g_{m_{Z}}$) is low, the miR-200/ZEB1 circuit will maintain the epithelial state (Left panel). When the basal production rate of ZEB1 mRNA is high, the miR-200/ZEB1 circuit can be bistable and allow epithelial state and mesenchymal state, but no hybrid E/M state can be acquired.

**Figure S2.** **Sensitivity analysis of miR-200/ZEB1/ESRP1/CD44 circuit driven by SNAIL for prostate cancer.** Alphanumeric codes on the x-axis represent the cases of different changed parameters. $\Delta S$ represents the change of the range of SNAIL levels for which the hybrid E/M exists. The red boxes highlight the cases where parameter perturbations have a relatively large effect on $\Delta S$.

Figure S2 (A-J) represent the cases for 20% increase and decrease in innate production rates and degradation rates of different species. A1 and A2 represent the case for 20% increase and decrease of miR-200 production rate (depicted by $g_{\mu_{200}}$). B1 and B2 represent the case for 20% increase and decrease of production rate of ZEB1 mRNA (depicted by $g_{m_{Z}}$). C1 and C2 represent the case for 20% increase and decrease of innate production rate of protein ZEB1 (depicted by $g_{Z}$). D1 and D2 represent the case for 20% increase and decrease of innate production rate of mRNA ESRP1 (depicted by $g_{m_{E}}$). E1 and E2 represent the case for 20% increase and decrease of innate production rate of ESRP1 protein (depicted by$g_{E}$). F1 and F2 represent the case for 20% increase and decrease of miR-200 degradation rate (depicted by $k_{\mu_{200}}$). G1 and G2 represent the case for 20% increase and decrease of innate degradation rate of ZEB1 mRNA (depicted by $k_{m_{Z}}$). H1 and H2 represent the case for 20% increase and decrease of innate degradation rate of protein ZEB1 (depicted by $k_{Z}$). I1 and I2 represent the case for 20% increase and decrease of innate degradation rate of ESRP1 mRNA (depicted by $k_{m_{E}}$). J1 and J2 represent the case for 20% increase and decrease of innate degradation rate of protein ESRP1 (depicted by $k_{E}$).

Figure S2 (K-Q) represent the cases for 20% increase and decrease in the threshold levels of the different shifted Hill functions. K1 and K2 represent the case for 20% increase and decrease in threshold levels of ZEB1 for miR-200 inhibition (depicted by $Z_{\mu_{200}}^{0}$). L1 and L2 represent the case for 20% increase and decrease in threshold levels of SNAIL for miR-200 inhibition (depicted by $S_{\mu_{200}}^{0}$). M1 and M2 represent the case for 20% increase and decrease in threshold levels of SNAIL for ZEB1 activation (depicted by $S_{m_{Z}}^{0}$). N1 and N2 represent the case for 20% increase and decrease in threshold levels of miR-200 (depicted by $\mu_{0}$). O1 and O2 represent the case for 20% increase and decrease in threshold levels of ZEB1 for ESRP1 inhibition (depicted by $Z_{m_{E}}^{0}$). P1 and P2 represent the case for 20% increase and decrease in threshold levels of ESRP1 for CD44 differential splicing (depicted by $E_{C_{s},0}$). Q1 and Q2 represent the case for 20% increase and decrease in threshold of differentially spliced CD44 for ZEB1 activation (depicted by $C_{s0,m_{Z}}$).

Figure S2 (R-W) represent the cases for 20% increase and decrease in fold-change parameters of different Hill functions. R1 and R2 represent the case for 20% increase and decrease in the fold change parameters for the inhibition of miR-200 by ZEB1 (depicted by $\lambda_{Z,\mu_{200}}$). S1 and S2 represent the case for 20% increase and decrease in fold change parameter for the inhibition of miR-200 by SNAIL (depicted by $\lambda_{S,\mu_{200}}$). T1 and T2 represent the case for 20% increase and decrease in fold change for the activation of ZEB1 by SNAIL (depicted by $\lambda_{S,m_{Z}}$). U1 and U2 represent the case for 20% increase and decrease in the fold change parameters for the inhibition of ESRP1 by ZEB1 (depicted by $\lambda_{Z,\mu_{200}}$). V1 and V2 represent the case for 20% increase and decrease in the fold change parameters for the activation of ZEB1 by differentially spliced CD44 (depicted by $\lambda_{C_{s},m_{Z}}$). W1 and W2 do not correspond with fold-changes, but represent the case for 20% increase and decrease in total levels of CD44 (depicted by $C_{T}$).

**Figure S3.** **The CD44s/ZEB1 feedback loop enables the tristability of the miR-200/ZEB1 circuit and the complete core regulatory circuit – miR-34/SNAIL/miR-200/ZEB1.** (**A**) The bifurcation diagram of ESRP1 mRNA in response to SNAIL levels for the miR-200/ZEB1 circuit with the CD44s/ZEB1 feedback loop. (**B**) The bifurcation diagram of ZEB1 mRNA levels in response to EMT-inducing signal ($S_{A}$) for the miR-34/SNAIL/miR-200/ZEB1 circuit with the CD44s/ZEB1 feedback loop. In (A) and (B), blue lines represent the levels of ESRP1 mRNA in stable states and right dotted lines represent the levels of ESRP1 mRNA in the unstable states. The corresponding phenotype to each stable state is labeled along with the blue line. The dotted arrows in (B) represent the transitions among E, E/M and M states.

**Figure S4. Immunofluorescence images showing different expression pattern of EMT markers in NSCLC cell lines – epithelial H820 cells, hybrid E/M H1975 cells and mesenchymal H1299 cells.** No Zeb1 expression is observed in the H820 (epithelial) cells and loss of membranous expression of CDH1 is shown in H1299 (mesenchymal) cells. H1975 (hybrid E/M) cells depict CDH1, Vim and Zeb1 at single cell level.

**Figure S5. mRNA levels of VIM in NSCLC cell lines.**

**
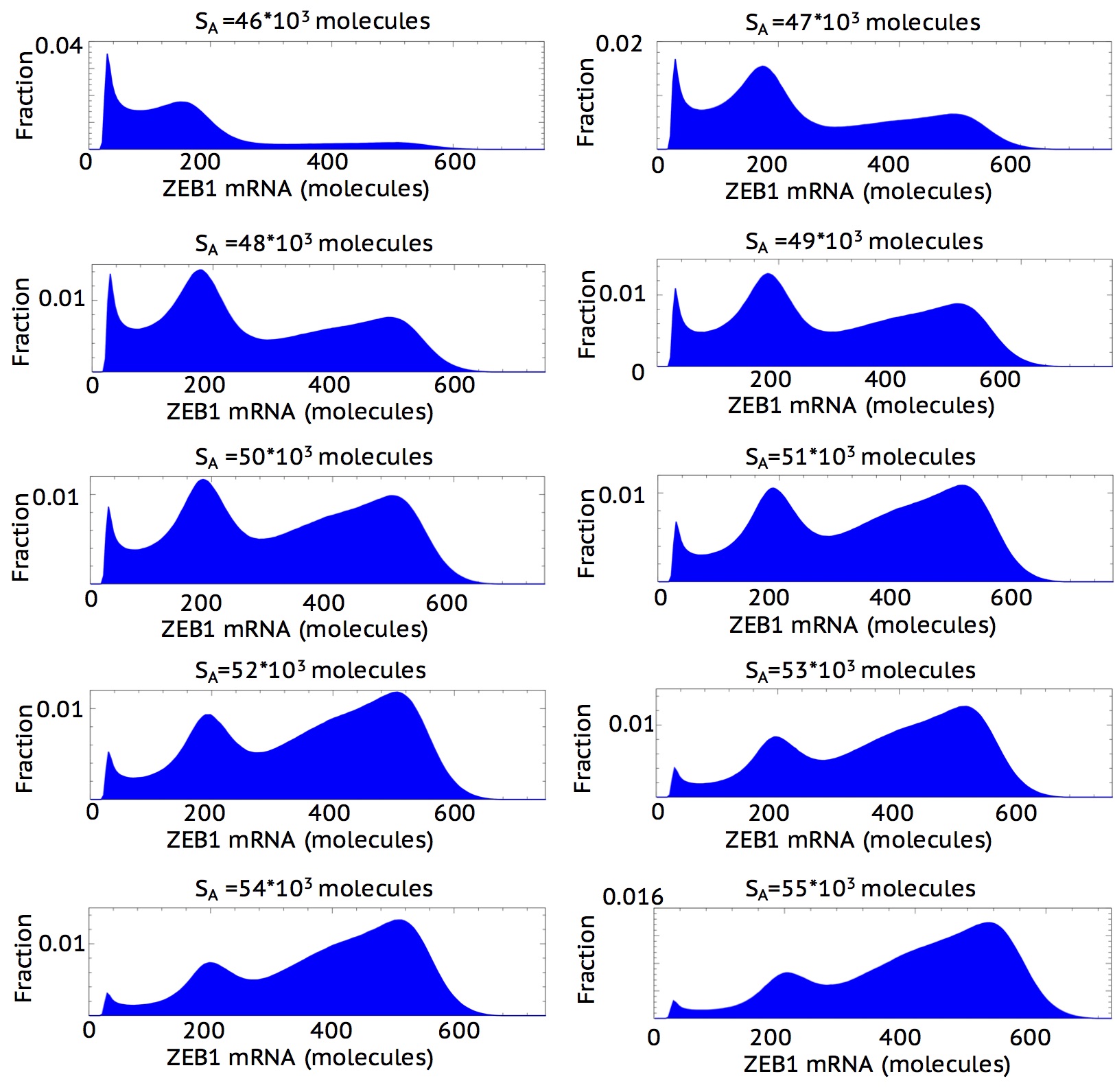
**

**Figure S6. Trimodal distribution of ZEB1 mRNA corresponding to the EMT-inducing signal** $\boldsymbol{S}_{\boldsymbol{A}}$ **in the TCS model on single-cell level.** Various levels of the EMT-inducing signal ($S_{A}$) can enable the tristability of the ZEB1 mRNA levels. The fraction of each group with ZEB1 levels low, intermediate and high can be adjusted by the levels of $S_{A}$.


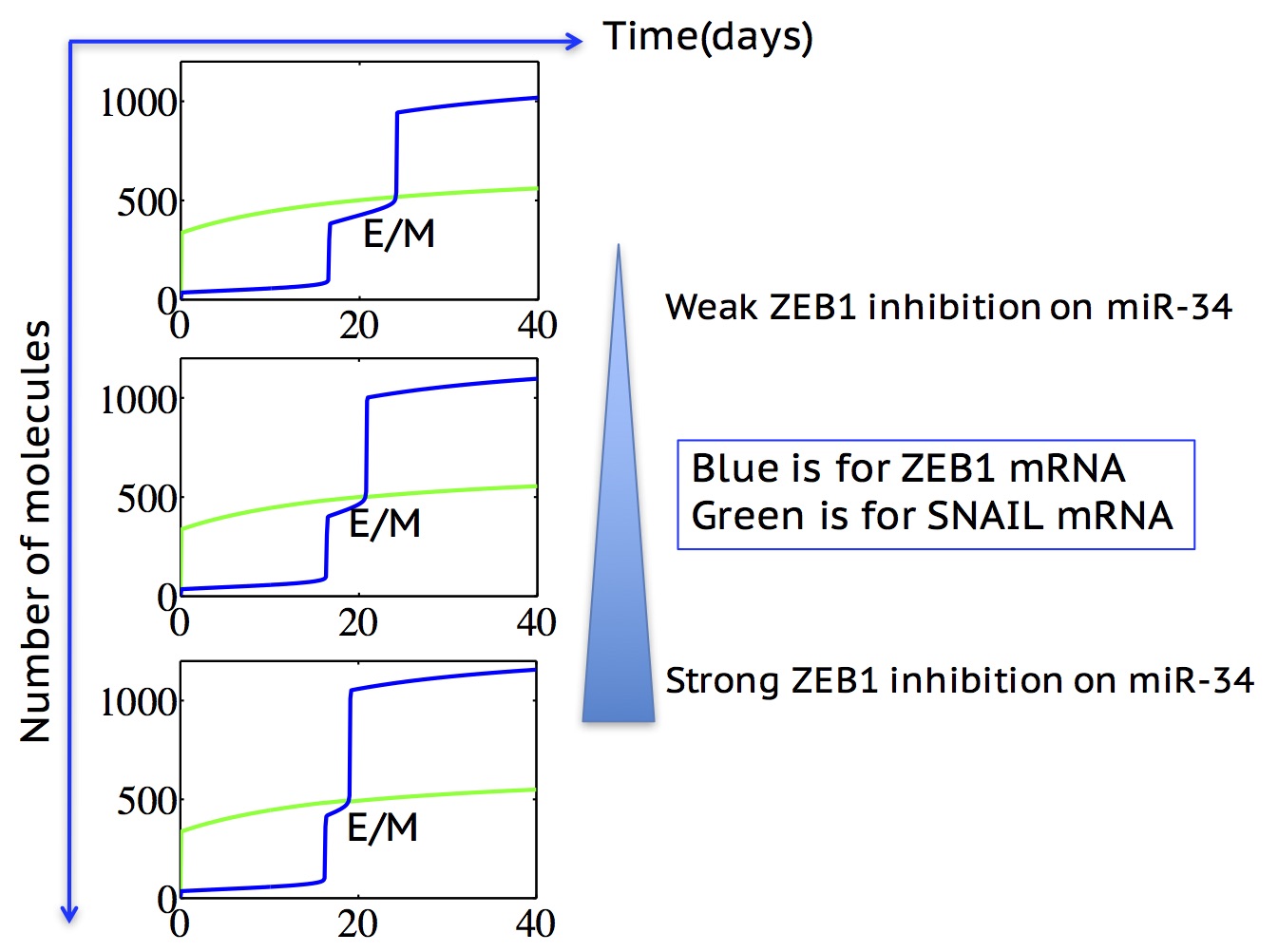


**Figure S7. Stronger inhibition of miR-34 by ZEB1 decrease the duration of the hybrid E/M phenotype during EMT.** The strength of ZEB1 inhibition on miR-34, as reflected by the value of the parameter $\lambda_{Z,\mu_{34}}$ in the TCS model, changed from 1 (no inhibition, top panel), 0.5 (intermediate inhibition, middle panel) to 0.2 (strong inhibition, down panel). In each figure, the x-axis represents the time – days and the y axis represent the levels of ZEB1 mRNA (blue curve) and SNAIL mRNA (green curve).

.


**Figure S8. Western blot analysis of EMT marker protein expression upon FOXC2 overexpression in HMLER cells.** Overexpression of FOXC2 doesn’t affect SNAIL but upregulate ZEB1, fibronection and vimentin and eliminate E-cadherin.

**Figure S9. Behaviors of the CBS model including transcription regulation by FOXC2.** (A) Including FOXC2 to the CBS framework – CBS-FOXC2. (B) Phase diagram of the $S_{A}$- $S_{I}$-driven CBS-FOXC2 circuit. $S_{A}$ represents an EMT-inducing signal on SNAIL and $S_{I}$ represents an imbibitional signal on FOXC2. Different colors in the phase diagram represent the different co-existences of stable states (phenotypes). (C) Bifurcation diagram of M marker (mesenchymal marker defined in the CBS model, which is proportional to the levels of SNAIL and ZEB1) in response to the EMT-inducing signal $S_{I}$ when there is no inhibition signal on FOXC2 (left panel, $S_{I}=0$) and there is inhibition on FOXC2 (right panel, $S_{I}=1.3\mu M$). Based on the CBS model, the CBS-FOXC2 circuit can still achieve the hybrid E/M state irrespective of the inhibition on FOXC2.

**Figure S10. SNAIL mRNA levels in response to the EMT-inducing signal** $\boldsymbol{S}_{\boldsymbol{A}}$ **when FOXC2 is inhibited (represented by** $\boldsymbol{S}_{\boldsymbol{I}}\boldsymbol{=2*}\boldsymbol{10}^{\boldsymbol{5}}$ **molecules).** The figure shows that SNAIL mRNA levels can be still upregulated by $S_{A}$ irrespective of the inhibition on FOXC2.

**Figure S11.** **ZEB1 self-activation promotes the irreversibility of the complete EMT.** The bifurcation diagram of ZEB1 mRNA levels in response to the EMT-inducing signal $S_{A}$ on SNAIL. From right to left, the strength of the ZEB1 self-activation gradually increase, as reflected by the increase of the value of the parameter $\lambda_{Z, m_{Z}}$ from 6, 6.5, 7, 7.5, 8, 8.5 to 9. Here the strength of ZEB1 inhibition on miR-34 is fixed and the parameter $\lambda_{Z, \mu_{34}}=0.2$.

**Figure S12.** **Evaluation of the effect of TWIST in regulating EMT by the TCS model. (A)** The EMT regulatory network integrating TWIST. TWIST functions as upstream activator of SNAIL (7,8). **(B)** The bifurcation diagram of ZEB mRNA levels in response to TWIST levels. The transcriptional activation of SNAIL by TWIST is represented by multiplying function $H^{S+}\left( T, T_{0,m_{S}},n_{T,m_{S},}\lambda_{T,m_{S}} \right)$ in the production term of the rate equation for SNAIL mRNA ($\frac{{dm}_{S}}{dt}$). $T$ represents the protein levels of TWIST, $T_{0,m_{S}}=100*{10}^{3}$ molecules, $n_{T,m_{S}}=2$ and $\lambda_{T,m_{S}}=9$.

**References**

1. Lu M, Jolly MK, Gomoto R, Huang B, Onuchic J, Ben-Jacob E. Tristability in Cancer-Associated MicroRNA-TF Chimera Toggle Switch. J Phys Chem B. 2013 Oct 24;117(42):13164–74.

2. Lu M, Jolly MK, Levine H, Onuchic JN, Ben-Jacob E. MicroRNA-based regulation of epithelial–hybrid–mesenchymal fate determination. Proc Natl Acad Sci. 2013 Nov 5;110(45):18144–9.

3. Zhang J, Tian X-J, Zhang H, Teng Y, Li R, Bai F, et al. TGF-β-induced epithelial-to-mesenchymal transition proceeds through stepwise activation of multiple feedback loops. Sci Signal. 2014 Sep 30;7(345):ra91.

4. Brown RL, Reinke LM, Damerow MS, Perez D, Chodosh L a., Yang J, et al. CD44 splice isoform switching in human and mouse epithelium is essential for epithelial-mesenchymal transition and breast cancer progression. J Clin Invest. 2011;121(3):1064–74.

5. Hollier BG, Tinnirello AA, Werden SJ, Evans KW, Taube JH, Sarkar TR, et al. FOXC2 expression links epithelial-mesenchymal transition and stem cell properties in breast cancer. Cancer Res. 2013 Mar 15;73(6):1981–92.

6. Werden SJ, Sphyris N, Sarkar TR, Paranjape AN, LaBaff AM, Taube JH, et al. Phosphorylation of serine 367 of FOXC2 by p38 regulates ZEB1 and breast cancer metastasis, without impacting primary tumor growth. Oncogene. 2016 Nov 17;35(46):5977-5988.

7. Smit MA, Geiger TR, Song J-Y, Gitelman I, Peeper DS. A Twist-Snail axis critical for TrkB-induced epithelial-mesenchymal transition-like transformation, anoikis resistance, and metastasis. Mol Cell Biol. 2009 Jul;29(13):3722–37.

8. Ip YT, Park RE, Kosman D, Yazdanbakhsh K, Levine M. dorsal-twist interactions establish snail expression in the presumptive mesoderm of the Drosophila embryo. Genes Dev. 1992 Aug;6(8):1518–30.
